# The Pressure Dependence of the Stability of the G-quadruplex Formed by d(TGGGGT)

**DOI:** 10.3390/life12050765

**Published:** 2022-05-21

**Authors:** Nabeel Tariq, Takuma Kume, Ujala N. Feroze, Robert B. Macgregor

**Affiliations:** Graduate Department of Pharmaceutical Sciences, Leslie Dan Faculty of Pharmacy, University of Toronto, Toronto, ON M5G1M2, Canada; nabeel.tariq@mail.utoronto.ca (N.T.); tkume@wisc.edu (T.K.); ujala.feroze@mail.utoronto.ca (U.N.F.)

**Keywords:** G-quadruplex, thermodynamics, hydrostatic pressure, volume, CD spectroscopy

## Abstract

The G-quadruplex (GQ), a tetrahelix formed by guanine-rich nucleic acid sequences, is a potential drug target for several diseases. Monomolecular GQs are stabilized by guanine tetrads and non-guanine regions that form loops. Hydrostatic pressure destabilizes the folded, monomolecular GQ structures. In this communication, we present data on the effect of pressure on the conformational stability of the tetramolecular GQ, d[5′-TGGGGT-3′]_4_. This molecule does not have loops linking the tetrads; thus, its physical properties presumably reflect those of the tetrads alone. Understanding the properties of the tetrads will aid in understanding the contribution of the other structural components to the stability of GQ DNA. By measuring UV light absorption, we have studied the effect of hydrostatic pressure on the thermal stability of the tetramolecular d[5′-TGGGGT-3′]_4_ in the presence of sodium ions. Our data show that, unlike monomolecular GQ, the temperature at which d[5′-TGGGGT-3′]_4_ dissociates to form the constituent monomers is nearly independent of pressure up to 200 MPa. This implies that there is no net molar volume difference (∆*V*) between the GQ and the unfolded random-coil states. This finding further suggests that the large negative ∆*V* values for the unfolding of monomolecular GQ are due to the presence of the loop regions in those structures.

## 1. Introduction

The guanine-quadruplex (GQ) is a secondary structure of DNA and RNA formed by molecules with sequences rich in guanines in the presence of some cations, such as sodium or potassium. The cation coordinates a tetrad of mutually hydrogen-bonded guanine residues that arrange in a plane, and these tetrads stack on one another to form the quadruplex. Intramolecular GQs form by the folding of a single DNA or RNA molecule, whereas intermolecular GQs contain two or four guanine-rich molecules [1,2,3]. Guanine-rich DNA sequences that form GQs in vitro are implicated in a number of human diseases and are situated in conserved regions in the genome across species [4,5,6,7,8,9,10,11]. The ability of GQs to self-assemble has led to their use in nanotechnology applications based on DNA [12,13,14].

Stability studies relying on changes in temperature, pH, and solvent parameters for GQ-containing solutions provide insight on the energetics of the folding process [15]. By studying the effect of pressure on GQ-containing systems, we can understand the role of water in the folding event [15,16,17,18,19]. The data to date show that the formation of monomolecular GQ structures is accompanied by a net release of water molecules into the bulk from the dehydration of metal cations and the formation of void volume by tetrad stacking [18,20,21]. The water-excluded voids, in turn, lead to a structure that is sensitive to pressure changes [15,22]. By analyzing the effect of pressure on the equilibrium between the folded and unfolded forms of the molecule, we can obtain the volumetric parameters that characterize the process [15,23,24,25]. Volumetric studies of GQs are important for understanding the hydration of these structures and the role the intracellular milieu may play in stabilizing or destabilizing these structures in vivo [26,27,28,29].

The effect of hydrostatic pressure on the conformational stability of monomolecular GQs has received some attention. In all cases, increasing the pressure leads to a destabilization of the folded structure to an oligonucleotide with no distinct secondary structure [15]. In addition, the role of the loops in the observed destabilization caused by pressure has also been investigated [21,30].

In this manuscript, we present data on the effect of hydrostatic pressure on the tetramolecular GQ formed by the hexanucleotide d[5′-TGGGGT-3′] (TG4T). The four-stranded quadruplex, d[5′-TGGGGT-3′]_4_ (TG4T-GQ), does not have any loop structures and only consists of four G-tetrads with single thymine residues at the 5′- and 3′- ends. Thus, the volumetric properties of this quadruplex should be representative of those of the G-tetrads that stabilize the structure. Our data show, rather surprisingly, that in contrast to the behaviour observed for monomolecular GQs, the thermal stability of the d[5′-TGGGGT-3′]_4_ structure is independent of pressure up to at least 200 MPa.

## 2. Results

### 2.1. Formation of TG4T-GQ

**Figure 1:**
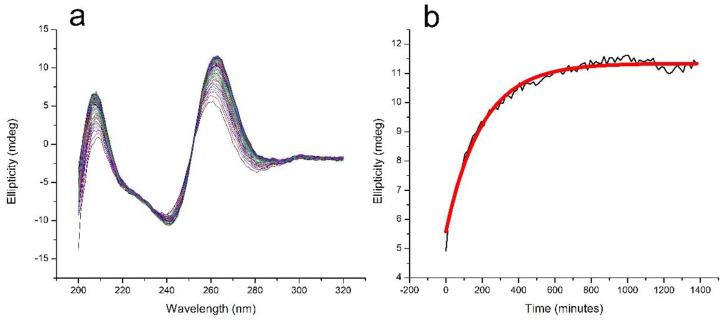
Kinetics of the formation of TG4T-GQ in aqueous solution containing 100 μM oligo and 1000 mM NaCl, 10 mM tris, pH 7.5. (**a**) The evolution of the CD spectrum of TG4T-GQ at 25 °C over time; a spectrum was acquired every 15 min for 23 h. (**b**) Plot of the ellipticity at 263 nm from (**a**) as a function of time, showing the approach to equilibrium in the formation of TG4T-GQ. The half-time of the formation process is approximately 140 min. It must be noted that these kinetic results are merely a means of estimating the time at which most of the sample has folded into a GQ to establish a starting point for our pressure experiments. The data points were fit to a simple exponential without consideration of the dead time or fourth-order reaction mechanism. Refer to the paper by Mergny and colleagues on tetramolecular kinetics for a more comprehensive kinetic analysis of four-stranded GQ [31].

The formation of TG4T-GQ from its constituent oligonucleotides is slow [31,32]. We monitored the formation of TG4T-GQ in a solution containing 100 μM oligo concentration and 100 mM NaCl at room temperature and observed continued formation up until the last day the sample was measured at 22 days (data not shown). To facilitate the formation of TG4T-GQ for the purposes of studying the pressure dependence of its thermal stability, we increased the NaCl concentration to 1000 mM. Figure 1a presents 92 circular dichroism (CD) spectra taken at 15-min intervals over 1380 min (23 h) to demonstrate the formation of the quadruplex. The maximum in the CD spectrum, at 263 nm, is characteristic of the formation of the four-stranded structure, and a plot of the intensity of the CD signal at 263 nm as a function of time is shown in Figure 1b. The signal at 263 nm leveled off by the end of this incubation period of 23 h. It is important to note the results in Figure 1b are qualitative in nature, as we fit them to a simple exponential without consideration of a fourth-order reaction mechanism. Rather than elucidate the kinetics parameters, we were interested in determining a starting point for our experiments.

### 2.2. Measurements at Elevated Pressures

**Figure 2:**
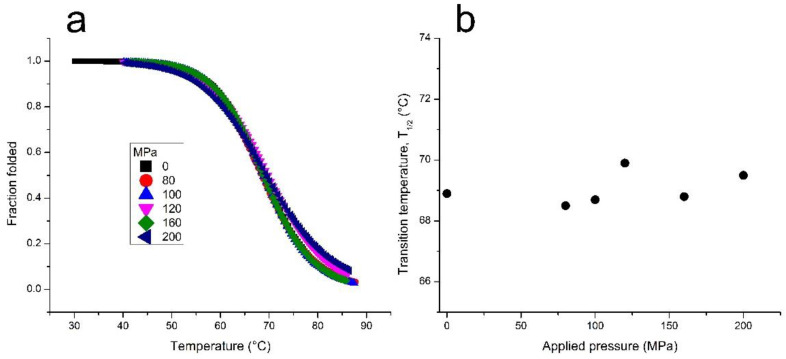
(**a**) Thermal denaturation of TG4T-GQ at different pressures. Fresh samples were used for each measurement. The samples contained 100 μM TG4T, 1000 mM NaCl, 10 mM tris, pH 7.5. (**b**) The pressure dependence of the melting temperature for the TG4T-GQ. The error in the *T*_1/2_ measurements is ±0.5 °C.

Next, we investigated the effect of pressure on the melting temperature (*T*_1/2_) of TG4T-GQ. Using the Clausius–Clapeyron equation, we calculated the volume change associated with the unfolding of the structure. According to the results shown in Figure 2 and Table 1, there was only a very modest change in the *T*_1/2_ of TG4T-GQ at pressures as high as 200 MPa. Based on these data, we calculated a molar volume change, Δ*V*, equal to −1.5 ± 2.3 cm^3^ mol^−1^ for the dissociation of TG4T-GQ into its four constituent oligonucleotides, d[5’-TGGGGT-3′]. Analysis of the enthalpy change upon dissociation assuming a single-step reaction is also reported in Table 1.

The four oligonucleotides in TG4T-GQ are oriented parallel to one another. We examined another parallel-stranded GQ formed from the oligo Pu22-T12T13, a variant of a 22-mer sequence proximal to the VEGF transcription initiation site [33,34]. We studied the dependence of the stability of the Pu22-T12T13-GQ on hydrostatic pressure. In comparison to the results for TG4T-GQ, the *T*_1/2_ of the parallel-stranded, monomolecular Pu22-T12T13-GQ showed significant destabilization with increasing pressure (Table 1). From our data, we calculated a molar volume change (Δ*V*) for the unfolding of Pu22-T12T13-GQ equal to −37.9 ± 10 cm^3^ mol*^−^*^1^.

## 3. Discussion

The number of studies that explore the effect of hydrostatic pressure on DNA G-quadruplex (GQ) structures is limited. In contrast to double-stranded conformations of nucleic acids, the G-quadruplexes have structural domains that may exhibit differential responses as a function of pressure. G-quadruplexes are stabilized by G-tetrads, which consist of four mutually hydrogen-bonded guanine residues that stack upon each other in the folded GQ structure. In monomolecular G-quadruplexes, successive G-tetrads are connected by nucleobases that are not guanine or cytosine residues. These bases that connect the stacked G-tetrads form the loops, which generally consist of two or three bases although loops with other numbers of bases are also possible. The bases in the loops do not form base-pairing interactions with other bases, and their existence leads to void volumes in folded G-quadruplex structures. The final component of the G-quadruplex structure are the cations that are coordinated by the O6 oxygen atoms of the guanine tetrads. When complexed with the G-tetrads, these ions are dehydrated, but upon denaturation of the G-quadruplex structure, they become rehydrated. The existence of structural voids and the rehydration of the cations released upon denaturation of the G-quadruplex structure are considered to be two of the primary causes of the destabilization of G-quadruplexes by pressure.

We can write the volume change arising from the denaturation of a monomolecular G-quadruplex as follows:

Δ*V*_(mmGQ)_ = Δ*V*_(tetrad)_ + Δ*V*_(loops)_ + Δ*V*_(ion)_

where Δ*V*_(mmGQ)_ is the observed volume change for a monomolecular G-quadruplex such as HTel (human telomeric sequence) or VEGF (vascular endothelial growth factor); Δ*V*_(tetrad)_ is the volume change that arises from the denaturation of the G-tetrads; Δ*V*_(loops)_ is the contribution of the loops to the observed value of Δ*V*_(mmGQ)_; and Δ*V*_(ion)_ is the volume change that arises from the rehydration of the ions coordinated by the O6 oxygen atoms of the G-tetrads from the native structure.

The role of the loops in the sensitivity of G-quadruplexes to pressure has been explored [21,30]. These studies show that the loops play an important role in the magnitude of the observed behaviour. Simple modifications to the loop regions result in significant changes in the unfolding volume of the monomolecular GQs formed by HTel and TBA (thrombin binding aptamer) GQs [15,21,30,35]. However, in all cases, regardless of the sequences of the loop, the volume change arising from the unfolding of the G-quadruplex is negative, i.e., the structure is destabilized by pressure. Upon the unfolding of the G-quadruplex structure, the bases that comprise the loops undergo a large change in the solvent-accessible surface area. In addition, the imperfect packing that arises from the structure of the loops in the folded structure leads to void volumes. These void volumes are lost upon the unfolding of the G-quadruplex structure.

To assess the contribution of the G-tetrads and ion release to the pressure dependence of the stability of G-quadruplexes, we have investigated the four-stranded complex formed by TG4T, which associates to form TG4T-GQ. The complex does not have loops; therefore, the observed volume change may be written as:

Δ*V*_(TG4T-GQ)_ = Δ*V*_(tetrad)_ + Δ*V*_(ion)_

where Δ*V*_(TG4T-GQ)_ is the observed volume change in the measurements carried out as a function of hydrostatic pressure. With knowledge of Δ*V*_(TG4T-GQ)_ and Δ*V*_(ion)_, we may then calculate the contribution of the G-tetrads to the measured volume change, Δ*V*_(TG4T-GQ)_.

TG4T-GQ has slow folding kinetics. Figure 1a,b show that in 1000 mM NaCl, a solution containing 100 μM TG4T formed over the course of 24 h with a half-time of formation of approximately 140 min. After incubating for 23 h, the CD spectrum was consistent with that of a G-quadruplex structure, and the spectra no longer changed with time. Because of the slow rate of formation of TG4T-GQ, each sample was subjected to only a single thermal denaturation.

When the TG4T-GQ samples were studied at different hydrostatic pressures, we did not observe a change in the stability of TG4T-GQ under the conditions of our measurements (see Figure 1b and Table 1). That is, we did not observe a statistically significant change in the *T*_1/2_ as a function of pressure (*p*) (i.e., Δ*T*_1/2_/Δ*p* ~0 °C/MPa). This result suggests that either (1) the volume change arising from rehydration of the ions has nearly the same magnitude but opposite sign from the volume change arising from the disruption of the G-tetrads, or (2) the volume change of both processes is negligible.

The denaturation of the G-tetrads entails the loss of the hydrogen bonds between the guanine residues, the unstacking of the G-tetrads, and the release of bound cations. The guanine–guanine hydrogen bonds lost upon denaturation will be replaced by hydrogen bonds with water. However, the unstacking of the G-tetrads will cause an increase in the solvent-accessible surface area (SASA). This change in SASA will involve exposure of the aromatic bases to water and a negative volume change. The release of the cations bound to the O6 oxygen atoms of the guanine residues will lead to the formation of interactions between the ions and water. This is also a process that proceeds with a negative volume change.

Thus, Δ*V*_(tetrad)_ and Δ*V*_(ion)_ are both anticipated to be negative. The observation that the *T*_1/2_ of TG4T-GQ is independent of hydrostatic pressure implies that Δ*V*_(tetrad)_ ≈ −Δ*V*_(ion)_. In the absence of loops, which give rise to void volumes, most of the effect of hydrostatic pressure on the stability of TG4T-GQ will arise from the changes in the interactions of water with the associated and dissociated states of this system. The properties of water, including its partial molar volume, are sensitive to temperature. For example, the partial molar volume passes from negative values at temperatures below ~50 °C to small positive values above this temperature [36]. From this, we infer that the interactions between the released ions and water are weaker in the higher temperature regime. Similar considerations apply to the interactions of the aromatic bases with water. Taken together, our findings suggest that possibility (2), the volume change of both processes is negligible, is relevant in this system, that is, the volume change arising from the hydration of the released ions and the exposed bases tend toward zero at the *T*_1/2_ of TG4T-GQ.

The tetramolecular G-quadruplex structure formed from TG4T has strands in an all-parallel conformation, whereas the monomolecular quadruplex formed from HTel is non-parallel. For this reason, we also present data on the effect of pressure on the conformational stability of the G-quadruplex formed by Pu22-T12T13 (Table 1). Pu22-T12T13 forms a monomolecular structure with parallel strand orientation. The data show that the molar volume change of unfolding this G-quadruplex is large and negative, similar to the other monomolecular structures that have been studied [15]. The results from Pu22-T12T13 and other parallel-stranded GQs from the literature demonstrate that the parallel strand orientation is not a cause for the near-zero Δ*V* of TG4T-GQ [37]. Thus, we conclude that the behaviour exhibited by TG4T-GQ is not related to the orientation of the strands.

Extending the present findings to the behaviour of single-stranded oligonucleotides that can form tetrahelical GQ conformations suggests that the pressure dependence of these monomolecular structures arises mostly from the differential hydration of the loops in these cases. It would be useful to explore the behaviour of TG4T-GQ stabilized by other ions or other tetramolecular G-quadruplexes, such as d([5′-TGGGT-3′])_4_ or d([5′-TGGGGGT-3′])_4_.

In conclusion, we present data that show that the thermal denaturation of the four-stranded G-quadruplex, TG4T-GQ, does not depend on pressure in solutions containing 1000 mM sodium chloride. We attribute the finding that, within experimental error, Δ*T*_1/2_/Δ*p* = 0 cm^3^ mol^−1^ to volume changes of both the disruption of the G-tetrads and the rehydration of the released cation tending toward zero at the temperature of the denaturation (~68 °C). This result suggests that the large, negative value of Δ*V* for the thermal denaturation of monomolecular G-quadruplex arises predominately from the presence of the loops in these structures and the void volumes to which they give rise.

## 4. Materials and Methods

We purchased the oligodeoxyribonucleotides TG4T (d[5′-TGGGGT-3′]) and Pu22-T12T13 (d[5′-CGGGGCGGGCCTTGGGCGGGGT-3′]) from ACGT (Toronto, ON, Canada). DNA was suspended in and dialyzed against Milli-Q^®^ ultra-purified water (Millipore Milli-Q Biocel Water Purification System, Sigma-Aldrich, Oakville, ON, Canada), vacuum-dried, and stored at −20 °C. DNA concentration was determined by measuring the absorbance at 260 nm using a molar extinction coefficient of 57,800 M^−1^ cm^−1^ for TG4T and 200,400 M^−1^ cm^−1^ for Pu22-T12T13 (Cary model 300 Bio spectrophotometer, Varian Canada, Inc., Mississauga, ON, Canada) [31,38,39,40]. Stock 100 mM tris(hydroxymethyl)aminomethane (tris, min. 99.5%, Bioshop, Burlington, ON, Canada) buffer adjusted to pH 7.5 with HCl was prepared, filter-sterilized (0.22 μM pore size), and diluted to 10 mM as the solvent for the TG4T and NaCl (1000 mM sodium chloride; Bioshop, Burlington, ON, Canada) solutions. TG4T-GQ was formed by heating the DNA samples dissolved in buffer to 95 °C for five minutes and then allowing the sample to cool to room temperature overnight in a 1-L Dewar flask fitted with a loose lid. Pu22-T12T13 was also heated and cooled overnight. Pu22-T12T13 was prepared in a solution of 2 mM KCl (potassium chloride, min 99.5%), 10 mM phosphoric acid, and 0.1 mM EDTA (ethylenediaminetetraacetic acid), titrated to pH 7.0 with TBAOH (tetrabutylammonium hydroxide), all purchased from Sigma-Aldrich, Oakville, ON, Canada. Note that 2 mM KCl was chosen because Pu220T12T13 formation is complete at this concentration [34].

Circular dichroism (CD) spectra were collected on a JASCO model J-1100 CD spectropolarimeter (Jasco, Easton, MD, USA) at 25 °C. The average of at least two scans are reported for each spectrum. The kinetics results in Figure 1 were prepared by first adding salt to the GQ at room temperature in a 1 mm quartz cuvette, placing the sample in the CD instrument, heating to 95 °C and holding for 5 min, then cooling to 25 °C at a rate of 1 °C/minute. The spectra were then collected at 15-min intervals for 23 h.

For the pressure experiments, a 350-mL sample cuvette was positioned in an optical high-pressure cell. Silicon oil was used as the pressure-transmitting liquid [41]. The light absorption of the sample in the pressure cell was monitored on a Uvikon model 860 spectrophotometer (Kontron, Inc., Everett, MA, USA). The temperature of the pressure cell was maintained by a brass thermal jacket with a programmable circulating water bath. The temperature was increased at 0.5 °C/min or 0.9 °C/minute, and the sample temperature was measured with a thermocouple inserted into the pressure cell. Instrument control and data acquisition were achieved using a Windows PC running a Python program. The stability of the G-quadruplex structure was observed at several static pressures between atmospheric pressure and 200 MPa. As described in previous work, we calculated the melting temperature (*T_m_*) as the mid-point of the normalized temperature-induced transition, and the enthalpy from the slope of the normalized transition using the van’t Hoff equation; this assumes that the unfolding is a single-step mechanism [42,43]. In the case of tetramolecular GQs, it is reported as the apparent melting temperature, *T*_1/2_ [31,44].

The molar volume change (Δ*V*) of the unfolding of GQs is calculated using the Clausius–Clapeyron equation,

$$\Delta V=\frac{\Delta H}{T_{m}}\;\frac{\Delta T_{m}}{\Delta p}$$

where Δ*H* is the change in enthalpy at the transition, and *p* is the hydrostatic pressure [15,37,45].

Throughout this manuscript, we refer to the single-stranded oligonucleotide, d[5′-TGGGGT-3′], as TG4T. The quadruplex formed from the association of four strands of TG4T, (d[5′-TGGGGT-3′])_4_, is referred to as TG4T-GQ.

## Figures and Tables

**Table 1:** The effect of hydrostatic pressure on the thermal denaturation of TG4T-GQ ^a^ and the G-quadruplex formed by Pu22-T12T13 ^b^.

GQ	Applied Pressure	*T*_1/2_ (°C) *	ΔH (kJ)	ΔV (cm^3^ mol^−1^)
TG4T	0	68.9	240 ± 14	−1.5 ± 2.3
	80	68.5	270 ± 16	
	100	68.7	300 ± 19	
	120	69.9	250 ± 19	
	160	68.8	280 ± 25	
	200	69.5	270 ± 18	
Pu22-T12T13	0	71.8	190 ± 23	−37.9 ± 10 **
	80	69.9	120 ± 19	
	180	60.7	130 ± 26	

^a^ TG4T samples: 100 μM TG4T, 1000 mM NaCl, 10 mM tris, pH 7.5. ^b^ Pu22-T12T13 samples: 100 µM Pu22-T12T13 in 2 mM KCl, 10 mM phosphoric acid, 0.1 mM EDTA, pH 7.0. * TG4T samples were heated at 0.9 °C/min, while Pu22-T12T13 samples were heated at 0.5 °C/min. Heating rates of 0.1, 0.5, and 0.9 °C/min did not influence the results for TG4T-GQ (unpublished). We present the data obtained with a 0.9 °C/min heating rate simply because it was the most complete set of data for TG4T. ** This error is an estimate based on prior publications with our pressure instrument [21].
